# Association of the CONUT score with mortality in critically ill patients with acute ischaemic stroke: a machine learning study with external validation

**DOI:** 10.3389/fneur.2026.1889258

**Published:** 2026-07-27

**Authors:** Ge Li, Wenshi Wei

**Affiliations:** Department of Neurology, Huadong Hospital Affiliated to Fudan University, Shanghai, China

**Keywords:** acute ischaemic stroke, CONUT score, intensive care unit, machine learning, malnutrition, mortality, prognosis, survival analysis

## Abstract

**Background:**

Malnutrition frequently occurs in patients with acute ischaemic stroke (AIS) and is associated with adverse clinical outcomes. Nevertheless, the prognostic utility of the Controlling Nutritional Status (CONUT) score in this population remains unclear. Therefore, we investigated the relationship between the CONUT score and mortality and developed a machine learning model for early risk prediction.

**Methods:**

Data from 1,103 patients with AIS in the MIMIC-IV database were retrospectively analyzed. An independent cohort comprising 659 patients from a Chinese tertiary hospital was included in the study. Survival analyses, multivariable Cox models, and subgroup analyses were conducted to evaluate the relationship between CONUT score and both 30-day and 365-day all-cause mortality. Machine learning models for 30-day mortality prediction were developed after Boruta-based feature selection in the training cohort, and model performance was evaluated by discrimination, calibration, decision analysis, and SHAP interpretation.

**Results:**

Elevated CONUT scores were independently associated with higher risks. After multivariable adjustment, a 1-unit increase in CONUT score corresponded to a 14.1% rise in short-term mortality risk and a 15.9% rise in long-term mortality risk. Kaplan–Meier curves further showed progressively reduced survival among patients with poorer nutritional status, and the associations remained stable across subgroup analyses. Among the six machine learning approaches, the CatBoost model achieved the strongest discriminative ability, yielding AUC values of 0.794 and 0.761 in the internal and external validation cohorts. Decision curve and calibration analyses also supported the robustness of the model.

**Conclusion:**

The CONUT score was independently associated with both short- and long-term mortality among critically ill AIS patients. The CatBoost-based prediction model showed favorable predictive performance in individualized risk assessment.

## 1 Introduction

Despite advances in reperfusion therapy and neurocritical care, stroke continues to impose a major public health burden worldwide (1, 2). The burden of acute ischaemic stroke (AIS) has further increased with population ageing and the growing prevalence of vascular comorbidities, especially in developing regions (1, 3). Beyond its high mortality, stroke is frequently associated with severe neurological impairment, prolonged hospitalization, and substantial healthcare expenditure (1, 4). Patients requiring intensive care represent a particularly vulnerable subgroup, often characterized by extensive neurological injury, multiple organ dysfunction, and increased susceptibility to complications (5). Although several prognostic indicators have been proposed for AIS, accurately identifying high-risk patients early after ICU admission remains a major clinical challenge (6). Therefore, there is a continued need for practical and reliable biomarkers that may support early risk assessment.

Nutritional status has emerged as an important yet frequently underrecognized factor influencing recovery and survival in patients with stroke (7, 8). Malnutrition is highly prevalent after AIS and may aggravate neurological damage through immune dysregulation, systemic inflammation, impaired tissue repair, and increased vulnerability to infection (9). Patients requiring intensive care are especially prone to nutritional deterioration because of dysphagia, impaired consciousness, hypercatabolic stress, and prolonged immobilization (10). Earlier research has reported that malnutrition is associated with higher mortality, poorer functional recovery, and longer hospital stays in stroke populations (11, 12). Consequently, early nutritional assessment has gained increasing attention in neurocritical care. Several evaluation instruments, including the Geriatric Nutritional Risk Index, Nutritional Risk Screening 2002, Mini Nutritional Assessment, and Subjective Global Assessment, have been investigated in stroke and critically ill populations (13, 14). However, many of these instruments rely partly on subjective evaluation or clinical information that may be difficult to obtain accurately in ICU settings. The Controlling Nutritional Status (CONUT) score, derived from total cholesterol, lymphocyte count, and serum albumin, provides a simple and objective measure of nutritional, inflammatory and immunological status based on routinely available laboratory parameters (15). Increasing evidence has linked elevated CONUT scores with adverse outcomes in cardiovascular disease, malignancy, and general stroke cohorts (16, 17). Nevertheless, evidence regarding its prognostic significance in patients with AIS remains limited, particularly with respect to long-term mortality and individualized risk prediction.

In the present study, we analyzed data from the MIMIC-IV database to explore the relationships between the CONUT score and both 30-day and 365-day mortality among patients with AIS. In addition, we developed several machine learning models for 30-day mortality prediction and externally validated the optimal model in an independent cohort of neurocritical patients from a tertiary hospital. By combining conventional survival analyses with machine learning techniques and external validation, this study sought to better characterize the prognostic relevance of nutritional status among patients with AIS and to provide a clinically applicable approach for individualized early prognostic evaluation.

## 2 Materials

### 2.1 Study population

The primary population was obtained from the MIMIC-IV database (18). As all patient records had been de-identified before release, informed consent was waived. An independent validation cohort was additionally collected from the neurological intensive care unit of a tertiary medical center in China and included consecutive patients admitted from January 2020 to January 2024. All clinical data were anonymized prior to analysis in accordance with ethical and privacy regulations. Ethical approval for the study was granted by Huadong Hospital (Approval No. 20260006).

**Figure 1:**
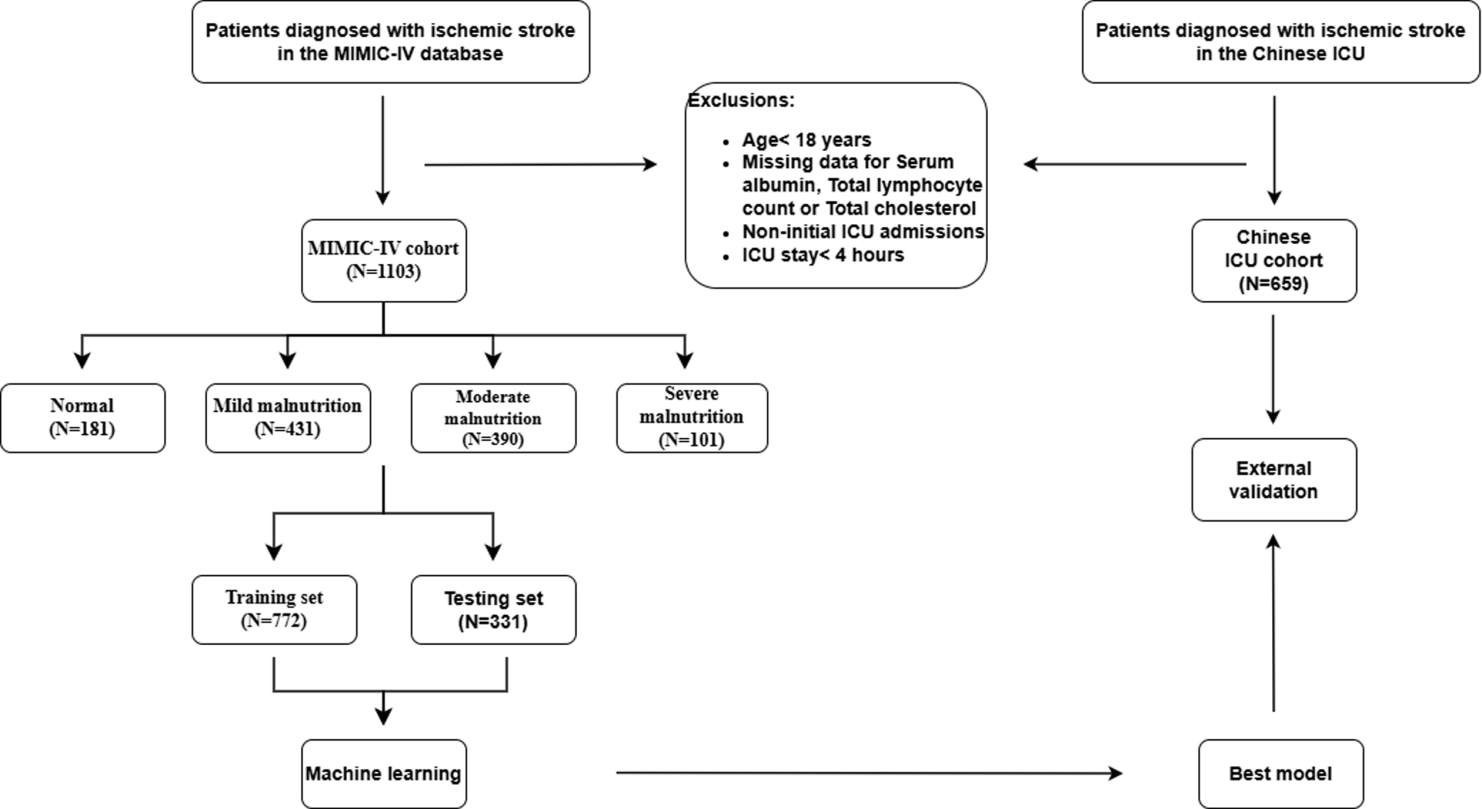
Flowchart of this study.

Patients with AIS were identified from the MIMIC-IV database according to ICD-9 and ICD-10 diagnostic codes. Patients were excluded according to the following criteria: (1) age younger than 18 years; (2) missing laboratory data required for calculation of the CONUT score, including total cholesterol, lymphocyte count, and serum albumin; (3) repeated ICU admissions; and (4) ICU length of stay shorter than 24 h. After screening, 1,103 eligible patients were included in the primary cohort. In addition, 659 patients from the Neurological ICU of Huadong Hospital were enrolled for external validation. According to the CONUT score, patients in the MIMIC-IV cohort were stratified into four nutritional groups: normal status, mild malnutrition, moderate malnutrition, and severe malnutrition. The detailed flowchart is presented in Figure 1.

### 2.2 Data extraction

Clinical information from the MIMIC-IV database was retrieved using Structured Query Language in accordance with a predefined study protocol (19). Demographic characteristics, severity scores, comorbidities, and laboratory findings were collected for all eligible patients. The CONUT score was calculated from total cholesterol, lymphocyte count, and serum albumin based on the scoring criteria shown in Table 1 (15). CONUT scores ranged from 0 to 12, with higher scores indicating poorer nutritional condition. According to the conventional classification criteria, patients were categorized as normal status (0–1 points), mild malnutrition (2–4 points), moderate malnutrition (5–8 points), and severe malnutrition (9–12 points).

**Table 1:** Definition and calculation of the CONUT score.

Parameters	Score			
Serum albumin (g/dL)	≥3.5	3.0–3.49	2.50–2.99	<2.5
Albumin score	0	2	4	6
Total cholesterol (mg/dL)	≥180	140–179	100–139	<100
Cholesterol score	0	1	2	3
Lymphocytes (count/mL)	≥1,600	1,200–1,599	800–1,199	<800
Lymphocyte score	0	1	2	3

To minimize the influence of therapeutic interventions during hospitalization, all variables were collected from the earliest records after ICU admission. Variables with more than 15% missing data were removed, whereas variables below this threshold were processed via mean imputation (20). Given the relatively low proportion of missing data among the retained variables, mean imputation was adopted as a simple and transparent approach to preserve the complete study cohort and maintain consistency across subsequent statistical and machine learning analyses.

### 2.3 Statistical analysis

Statistical analyses were conducted using R software and SPSS Statistics. Continuous variables were summarized as mean ± standard deviation or median with interquartile range based on their distribution patterns, whereas categorical variables were presented as frequencies and proportions. Continuous variables were compared using one-way analysis of variance or the Kruskal–Wallis test, depending on data distribution. Categorical variables were analyzed using the chi-square test or Fisher’s exact test. Statistical significance was defined as a two-tailed *p* value < 0.05.

Kaplan–Meier analysis was used to compare 30-day and 365-day survival across the four CONUT-defined nutritional groups. Survival curves were compared using the log-rank test. Cox proportional hazards models were then applied to examine the relationships between CONUT score and mortality outcomes. Hazard ratios and 95% confidence intervals were estimated. Two regression models were constructed: Model 1 without adjustment and Model 2 adjusted for potential confounding factors that were selected *a priori* based on their established clinical relevance and evidence from previous studies, including age, sex, atrial fibrillation, diabetes mellitus, hypertension, platelet count, red blood cell count, white blood cell count, blood urea nitrogen, serum creatinine, blood pressure, heart rate, and SOFA score. Subgroup analyses stratified by sex, age, hypertension, atrial fibrillation, and diabetes mellitus were additionally performed to assess the consistency of the associations, and interaction tests were conducted accordingly.

Machine learning analyses were performed to develop prediction models for 30-day all-cause mortality in patients with AIS. The MIMIC-IV cohort was first randomly separated into training and internal validation cohorts in a 7:3 proportion, while the independent cohort was used for external validation. The Boruta algorithm was subsequently used for feature selection only in the training cohort, and 12 important variables were retained for model development. To improve model robustness and reduce the risk of overfitting, five-fold cross-validation was performed during model development within the training cohort. Six machine learning algorithms were constructed and evaluated, including CatBoost, LightGBM, random forest, multilayer perceptron, logistic regression, and XGBoost. Predictive performance was evaluated based on receiver operating characteristic curves and area under the curve values. The optimal model identified in the internal validation cohort was subsequently tested in the external cohort. Decision curve analysis (DCA), calibration curves and the Brier score were further performed to evaluate model calibration and clinical utility. SHAP analysis was additionally used to interpret the contribution of individual features to model predictions.

## 3 Results

### 3.1 Baseline characteristics

1,103 patients with AIS from the MIMIC-IV database were ultimately enrolled in the study. Baseline characteristics stratified according to CONUT categories are presented in Table 2. The median age of the cohort was 70 years, and 52.31% were male. The proportion of male patients was highest in the severe malnutrition group (62.38%). Significant differences in laboratory findings and disease severity were identified across the four nutritional categories. Relative to the other nutritional groups, those with severe malnutrition generally exhibited lower levels of white blood cell count, platelet count, red blood cell count, and haemoglobin. In contrast, blood urea nitrogen, serum creatinine, blood glucose, and SOFA scores were markedly elevated in the severe malnutrition group, suggesting more severe physiological impairment and organ dysfunction. In addition, both 30-day and 365-day mortality rates increased progressively with worsening nutritional status and were highest in the severe malnutrition group.

**Table 2:** Baseline characteristics of AIS patients stratified by CONUT score.

Variable	Overall (*N* = 1,103)	Normal (*N* = 181)	Mild malnutrition (*N* = 431)	Moderate malnutrition (*N* = 390)	Severe malnutrition (*N* = 101)	*p*-value
Age (years)	70.00 (60.00–81.00)	64.00 (54.00–75.00)	71.00 (61.00–83.00)	73.00 (62.00–83.00)	69.00 (60.00–77.00)	<0.001
Sex: men (n, %)	577.00 (52.31%)	94.00 (51.93%)	219.00 (50.81%)	201.00 (51.54%)	63.00 (62.38%)	0.204
WBC (K/uL)	10.40 (7.90–13.70)	10.80 (8.50–13.40)	10.30 (8.00–13.40)	10.40 (7.70–14.00)	10.10 (6.30–14.70)	0.571
RBC (m/uL)	4.01 (3.46–4.51)	4.43 (4.01–4.73)	4.11 (3.61–4.64)	3.82 (3.21–4.26)	3.33 (2.86–3.91)	<0.001
Platelet (K/uL)	204.00 (155.00–264.00)	224.00 (186.00–271.00)	204.00 (164.00–260.00)	197.00 (147.00–268.00)	172.00 (114.00–262.00)	<0.001
Neutrophil (K/uL)	8.08 (5.76–11.38)	7.60 (5.36–9.83)	8.04 (5.89–11.07)	8.40 (5.92–12.06)	8.86 (5.83–13.33)	0.017
Triglycerides (mg/dl)	114.00 (79.00–153.00)	114.00 (89.00–158.00)	114.00 (77.00–151.00)	107.00 (77.00–148.18)	127.00 (86.00–173.00)	0.049
Lymphocyte (K/uL)	1.19 (0.78–1.77)	1.98 (1.66–2.52)	1.24 (0.91–1.77)	0.95 (0.62–1.34)	0.69 (0.48–1.02)	<0.001
Hemoglobin (g/dL)	11.90 (10.20–13.40)	13.20 (11.90–14.20)	12.30 (10.90–13.60)	11.10 (9.50–12.80)	9.80 (8.50–11.20)	<0.001
Scr (mg/dl)	1.00 (0.70–1.30)	0.90 (0.70–1.10)	0.90 (0.70–1.20)	1.00 (0.80–1.50)	1.10 (0.80–1.80)	<0.001
BUN (mg/dl)	18.00 (13.00–27.00)	14.00 (11.00–18.00)	17.00 (12.00–23.00)	21.00 (14.00–33.00)	25.00 (15.00–44.00)	<0.001
PT (sec)	13.00 (11.90–14.60)	12.10 (11.50–13.00)	12.70 (11.80–14.10)	13.50 (12.30–15.70)	15.20 (13.70–17.80)	<0.001
Glucose (mg/dl)	127.00 (104.00–164.00)	115.00 (97.00–139.00)	126.00 (104.00–166.00)	133.00 (105.00–171.00)	135.00 (108.00–177.00)	<0.001
Sodium (mmol/L)	139.00 (137.00–142.00)	140.00 (138.00–142.00)	140.00 (137.00–142.00)	139.00 (136.00–142.00)	139.00 (135.00–142.00)	0.038
Potassium (mmol/L)	4.00 (3.70–4.40)	4.00 (3.70–4.30)	4.00 (3.70–4.40)	4.00 (3.60–4.50)	4.00 (3.70–4.60)	0.923
HDL (mg/dl)	43.00 (33.00–55.00)	49.00 (41.00–61.00)	47.00 (37.00–60.00)	39.00 (30.00–50.00)	29.00 (20.00–42.00)	<0.001
TC (mg/dl)	151.00 (124.00–185.00)	192.00 (166.00–216.00)	161.00 (137.00–190.00)	132.00 (107.00–157.00)	102.00 (85.00–128.00)	<0.001
ALT (U/L)	21.00 (14.00–37.00)	18.00 (13.00–26.00)	21.00 (14.00–34.00)	23.00 (14.00–43.00)	21.00 (14.00–45.00)	0.005
AST (U/L)	28.00 (19.00–47.00)	22.00 (18.00–31.00)	27.00 (20.00–43.00)	32.00 (20.00–58.00)	39.00 (22.00–81.00)	<0.001
CONUT, median (p25 - p75)	4.00 (2.00–6.00)	1.00 (0.00–1.00)	3.00 (2.00–4.00)	6.00 (5.00–7.00)	9.00 (9.00–11.00)	<0.001
GCS	14.00 (11.00–15.00)	14.00 (12.00–15.00)	14.00 (11.00–15.00)	14.00 (10.00–15.00)	15.00 (12.00–15.00)	0.033
SOFA	3.00 (2.00–5.00)	2.00 (1.00–3.00)	3.00 (2.00–5.00)	4.00 (2.00–7.00)	6.00 (4.00–9.00)	<0.001
SIRS	2.00 (2.00–3.00)	2.00 (2.00–3.00)	2.00 (1.00–3.00)	3.00 (2.00–3.00)	3.00 (2.00–3.00)	<0.001
HR (bpm)	82.00 (72.00–95.00)	79.00 (70.00–88.00)	79.00 (70.00–92.00)	85.50 (74.00–98.00)	89.00 (78.00–106.00)	<0.001
SBP (mmHg)	136.05 (119.00–153.00)	141.00 (126.00–159.00)	139.00 (122.00–155.00)	133.50 (114.00–149.00)	122.00 (102.00–140.00)	<0.001
DBP (mmHg)	76.00 (65.00–88.00)	77.00 (70.00–91.00)	77.29 (66.00–89.00)	74.00 (62.00–86.00)	68.00 (57.00–82.00)	<0.001
RR (breaths/min)	19.00 (16.00–22.00)	18.00 (16.00–22.00)	18.00 (16.00–22.00)	19.00 (16.00–23.00)	21.00 (17.00–25.00)	<0.001
Diabetes (n, %)	361.00 (32.73%)	51.00 (28.18%)	122.00 (28.31%)	150.00 (38.46%)	38.00 (37.62%)	0.006
Arterial fibrillation (*n*, %)	414.00 (37.53%)	41.00 (22.65%)	151.00 (35.03%)	178.00 (45.64%)	44.00 (43.56%)	<0.001
Hypertension (*n*, %)	591.00 (53.58%)	124.00 (68.51%)	250.00 (58.00%)	175.00 (44.87%)	42.00 (41.58%)	<0.001
30-day mortality (*n*, %)	148.00 (13.42%)	10.00 (5.52%)	49.00 (11.37%)	62.00 (15.90%)	27.00 (26.73%)	<0.001
365-day mortality (*n*, %)	195.00 (17.68%)	11.00 (6.08%)	63.00 (14.62%)	83.00 (21.28%)	38.00 (37.62%)	<0.001

### 3.2 Associations between the CONUT score and outcome events

**Figure 2:**
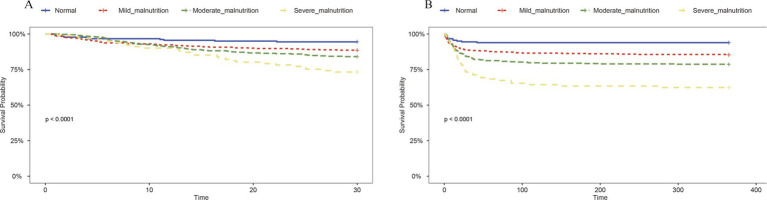
Kaplan–Meier curves for 30-day **(A)** and 365-day **(B)** all-cause mortality according to CONUT categories.

Kaplan–Meier survival curves evaluated survival among the four CONUT-defined nutritional groups (Figure 2). Marked differences in both 30-day and 365-day survival were observed (*p* < 0.001). Survival probability progressively decreased with increasing CONUT scores. Patients in the normal nutritional group showed the most favorable survival outcomes, whereas those with severe malnutrition exhibited the poorest prognosis and the steepest decline in survival over time.

Cox regression analyses further demonstrated significant associations between higher CONUT scores and higher risks of short- and long-term mortality (Table 3). When analyzed as a continuous variable, each additional point in CONUT score was linked to a 14.1% higher risk of 30-day mortality (Model 2: HR = 1.141, 95% CI: 1.072–1.214, *p* < 0.001) and a 15.9% higher risk of 365-day mortality (Model 2: HR = 1.159, 95% CI: 1.098–1.224, p < 0.001). Similar results were obtained after stratification by nutritional status. Compared with the normal nutrition group, moderate and severe malnutrition were linked to markedly increased mortality risk. In the fully adjusted model, patients with severe malnutrition had a 4.303-fold higher risk of 30-day mortality (95% CI: 1.963–9.433, *p* < 0.001) and a 4.872-fold higher risk of 365-day mortality (95% CI: 2.373–10.003, p < 0.001).

**Table 3:** Cox regression analyses of the associations between CONUT score and all-cause mortality.

Categories	Model 1			Model 2		
	HR (95% CI)	*p*-value	P for trend	HR (95% CI)	*p*-value	P for trend
30-day mortality						
Continuous variable	1.157 (1.097–1.221)	<0.001		1.141 (1.072–1.214)	<0.001	
Group			<0.001			<0.001
Normal (*N* = 181)	Ref					
Mild malnutrition (*N* = 431)	2.129 (1.078–4.202)	0.029		1.963 (0.985–3.91)	0.055	
Moderate malnutrition (*N* = 390)	2.991 (1.533–5.832)	<0.001		2.467 (1.222–4.978)	0.012	
Severe malnutrition (*N* = 101)	5.207 (2.52–10.757)	<0.001		4.303 (1.963–9.433)	<0.001	
365-day mortality						
Continuous variable	1.192 (1.138–1.249)	<0.001		1.159 (1.098–1.224)	<0.001	
Group			<0.001			<0.001
Normal (*N* = 181)	Ref					
Mild malnutrition (*N* = 431)	2.513 (1.325–4.769)	0.005		2.16 (1.13–4.13)	0.020	
Moderate malnutrition (*N* = 390)	3.742 (1.995–7.018)	<0.001		2.825 (1.465–5.445)	0.002	
Severe malnutrition (*N* = 101)	7.076 (3.616–13.846)	<0.001		4.872 (2.373–10.003)	<0.001	

Model 1 without adjustment. Model 2 was adjusted for age, sex, atrial fibrillation, diabetes mellitus, hypertension, platelet count, red blood cell count, white blood cell count, blood urea nitrogen, serum creatinine, blood pressure, heart rate, and SOFA score.

### 3.3 Subgroup analysis

**Figure 3:**
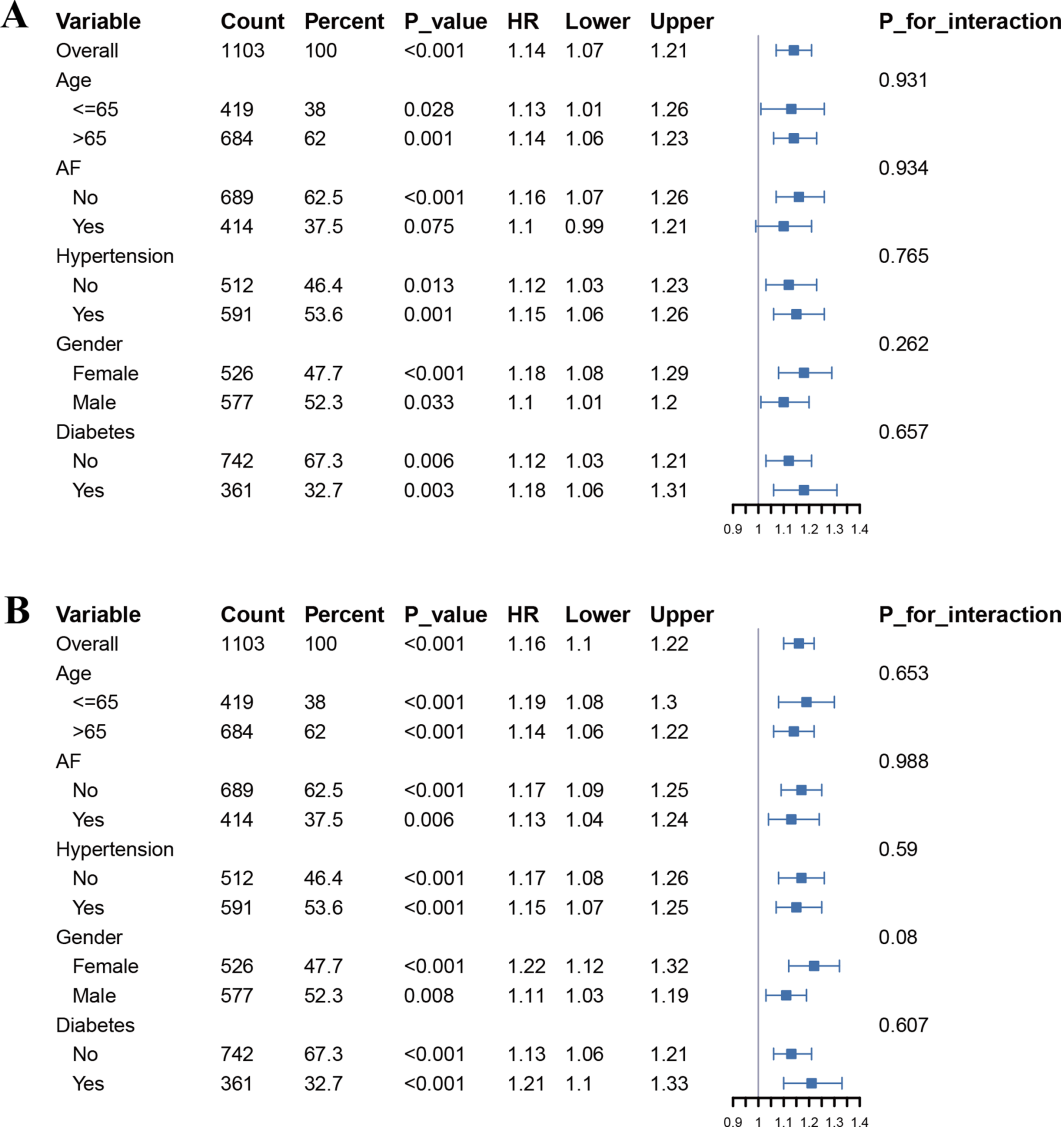
Subgroup analyses of the association between CONUT score and 30-day **(A)** and 365-day **(B)** all-cause mortality.

Subgroup analyses were conducted according to sex, age, atrial fibrillation, diabetes mellitus, and hypertension to assess the stability of the association between CONUT score and mortality risk (Figure 3). Higher CONUT scores were consistently linked to higher risks of all-cause mortality across nearly all subgroups. No significant interactions were detected between CONUT score and subgroup variables (all P for interaction > 0.05), implying that the prognostic value of the CONUT score remained stable among different patient populations. These results further support the robustness of the relationship between poor nutritional status and adverse outcomes in critically ill patients with AIS.

### 3.4 Model development and validation

**Figure 4:**
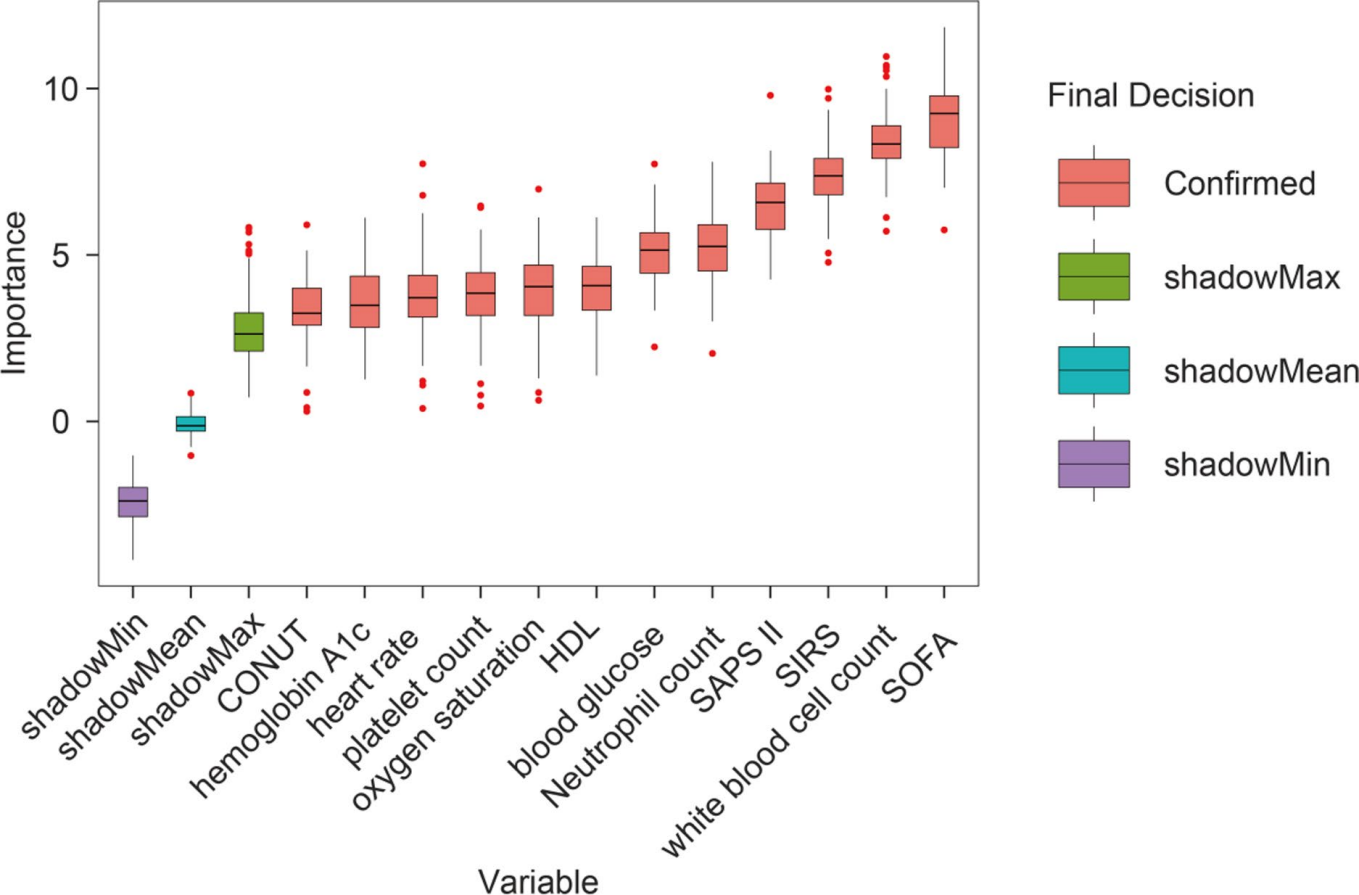
Feature selection.

The Boruta algorithm was applied to identify important features, and the results are presented as a variable importance boxplot (Figure 4). By comparing the importance of candidate variables with randomly generated shadow features (shadowMin, shadowMean, and shadowMax), Boruta identified 12 confirmed variables for subsequent model construction. These included CONUT score, platelet count, high-density lipoprotein cholesterol, heart rate, hemoglobin A1c, SAPS II score, white blood cell count, oxygen saturation, blood glucose, Neutrophil count, SIRS score, and SOFA score. The importance values of all selected variables were significantly higher than shadowMax, indicating that these features played major roles in predicting prognosis in patients with AIS. Five-fold cross-validation demonstrated stable model performance during model development.

**Figure 5:**
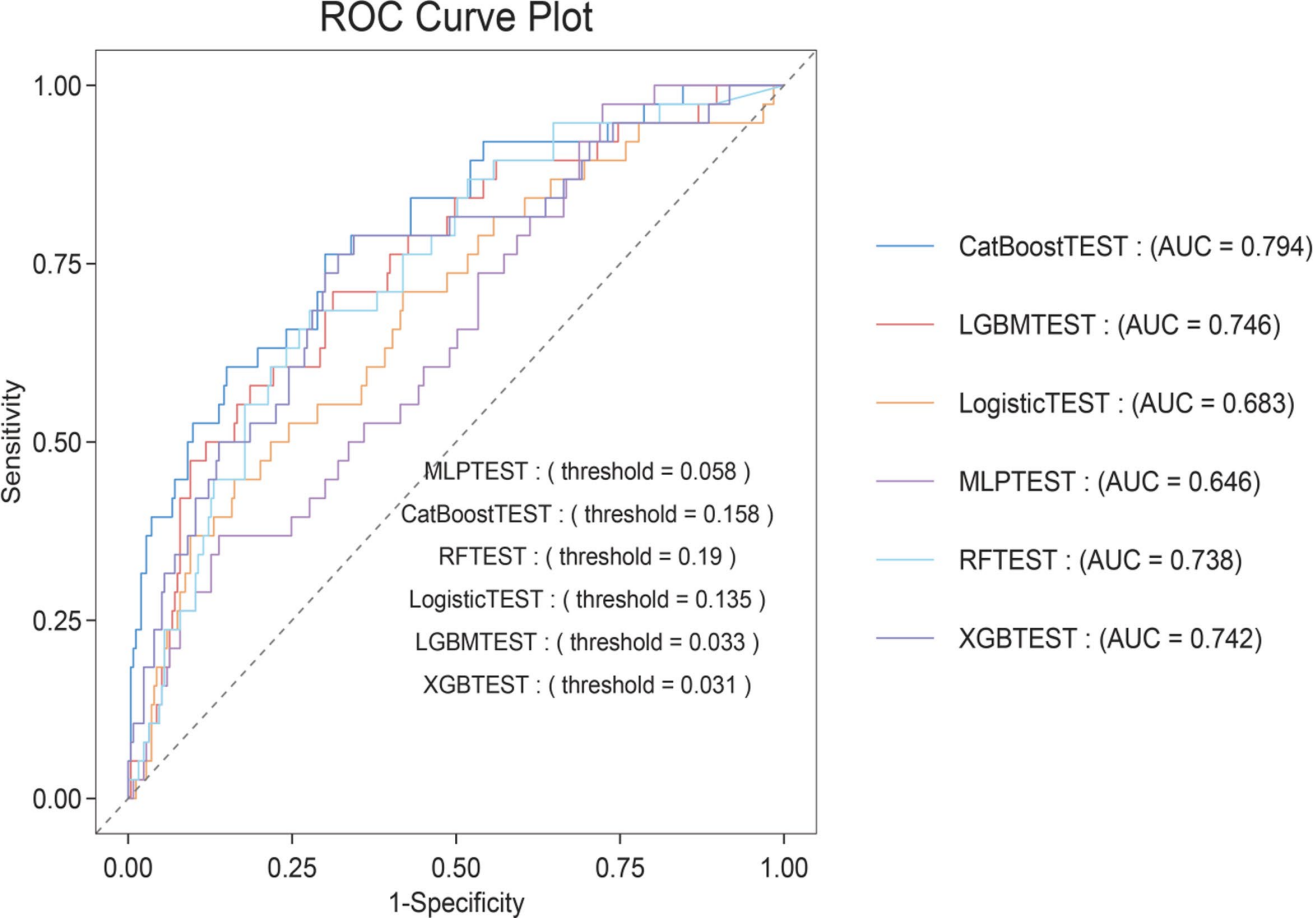
ROC curves of six machine learning models for predicting 30-day mortality in the internal validation cohort.

The MIMIC-IV cohort was randomly split into training and internal validation sets at a ratio of 7:3. Using the variables selected by the Boruta algorithm, six machine learning models were constructed to predict 30-day all-cause mortality in patients with acute ischaemic stroke, including CatBoost, LightGBM, logistic regression, multilayer perceptron, random forest, and XGBoost. Figure 5 presents the ROC curves of the six machine learning models. Among these models, CatBoost showed the highest predictive accuracy, with an AUC of 0.794. The corresponding AUC values for LightGBM, logistic regression, multilayer perceptron, random forest, and XGBoost were 0.746, 0.683, 0.646, 0.738, and 0.742, respectively. Overall, the CatBoost model demonstrated the strongest discriminative ability for estimating 30-day mortality in patients with AIS.

**Figure 6:**
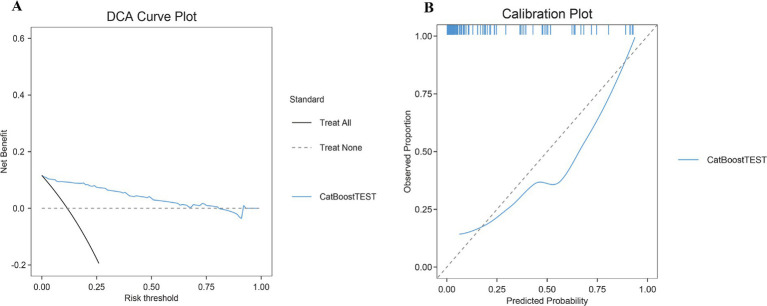
Decision curve analysis **(A)** and calibration curve **(B)** of the CatBoost model for predicting 30-day mortality.

Further evaluation of the CatBoost model was performed by calibration curves, the Brier score and decision curve analysis (Figure 6). The calibration curve showed strong consistency, with the fitted curve remaining close to the ideal reference line, indicating satisfactory calibration ability of the model. The model achieved a Brier score of 0.09, indicating satisfactory calibration performance. DCA indicated that the CatBoost model achieved greater net clinical benefit than the “treat-none” and “treat-all” approaches. These findings suggest that the model may support meaningful clinical value for risk prediction in patients with AIS.

**Figure 7:**
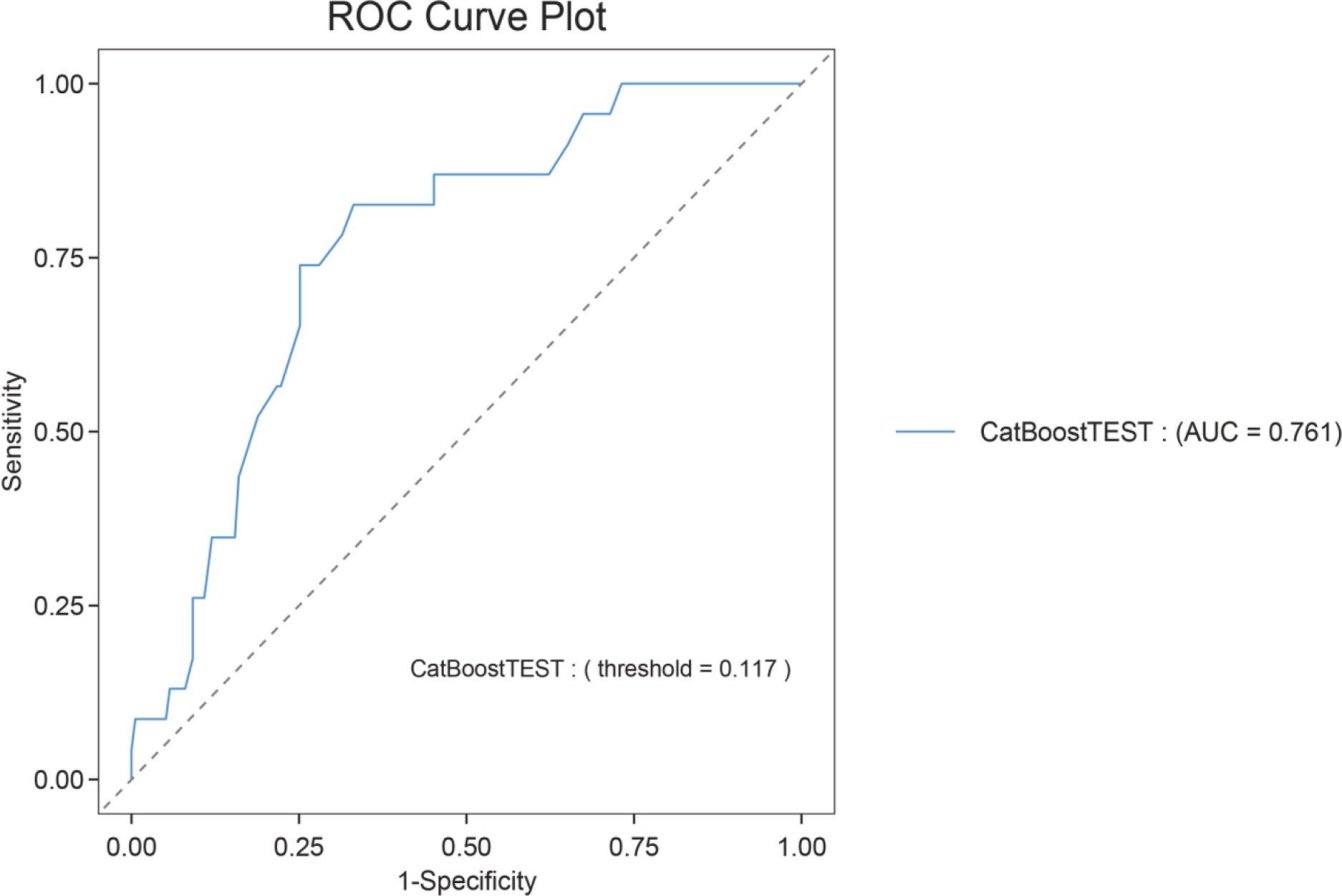
ROC curve of the CatBoost model in the external validation cohort.

To further assess the generalizability of the optimal model, an independent cohort of 659 patients with AIS from a Chinese tertiary hospital was used for external validation. The ROC curve for predicting 30-day all-cause mortality is shown in Figure 7. In this cohort, the CatBoost model yielded an AUC of 0.761, indicating that the model maintained good discriminative performance and predictive stability in an independent real-world clinical population.

### 3.5 Model explainability

**Figure 8:**
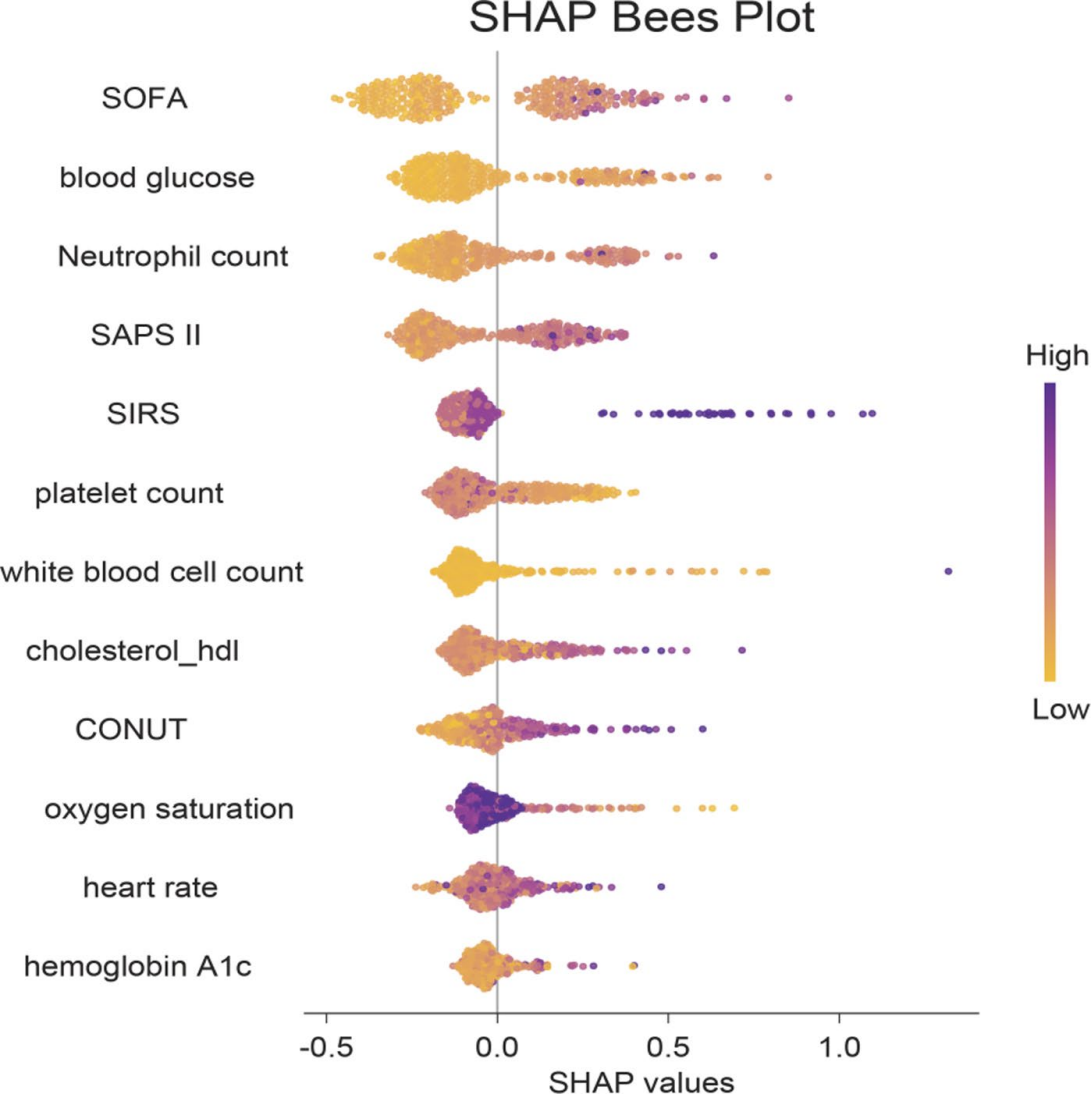
SHAP summary plot of the CatBoost model.

SHAP analysis was further conducted to improve interpretation of the CatBoost model and to evaluate the contribution of individual variables to 30-day mortality prediction (Figure 8). The SHAP summary plot demonstrated that hemoglobin A1c, heart rate, oxygen saturation, CONUT score, and HDL cholesterol were major contributors to model output. Higher CONUT scores, increased white blood cell count, elevated heart rate, and higher SOFA scores were generally associated with higher mortality risk, whereas higher oxygen saturation and platelet count tended to contribute to lower predicted risk. Overall, the SHAP results highlighted the combined importance of nutritional status, systemic inflammation, metabolic disturbance, and disease severity to prognosis in critically ill patients with AIS.

## 4 Discussion

We found that elevated CONUT scores were independently linked to higher risks of both 30-day and 365-day mortality in patients with AIS. Kaplan–Meier and multivariable Cox analyses consistently demonstrated that worsening nutritional status was linked to poorer survival outcomes, and these associations remained stable across subgroup analyses. In addition, we established and externally validated a machine learning model for early mortality prediction, with the CatBoost model showing the best predictive performance. Compared with logistic regression, the CatBoost model achieved better discriminative performance, supporting its potential utility for individualized risk prediction.

Earlier studies have similarly found that higher CONUT scores are linked to worse outcomes after stroke (16, 21). Naito et al. showed that elevated CONUT scores were associated with unfavorable functional recovery after acute ischaemic stroke (21). Later studies further suggested that malnutrition was linked to higher mortality and disability in stroke patients (16). Compared with previous research, our study specifically focused on critically ill AIS patients and additionally incorporated machine learning and external validation, which may provide more practical value for early risk stratification.

The mechanisms linking higher CONUT scores to poor outcomes after acute ischaemic stroke are likely multifactorial. First, serum albumin, an important component of the CONUT score, reflects both nutritional condition and systemic inflammatory activity (22). Hypoalbuminemia is common in critically ill stroke patients and has been linked to increased blood–brain barrier disruption, oxidative stress, and cerebral edema (23). Experimental studies have also suggested that albumin may exert neuroprotective effects through antioxidant activity and maintenance of microvascular perfusion (24). Therefore, reduced albumin levels may contribute to secondary brain injury and worse neurological recovery after stroke.

Lymphocyte count represents another key component of the CONUT score. Acute stroke can induce a state of immunosuppression characterized by lymphocyte depletion and impaired cellular immunity, which increases susceptibility to pneumonia, urinary tract infection, and other complications during hospitalization (25, 26). Previous studies have shown that stroke-associated immune dysfunction is closely related to unfavorable outcomes and increased mortality (26, 27). In addition, low cholesterol levels may reflect chronic malnutrition, frailty, and insufficient metabolic reserve (26). Cholesterol also plays an important role in cell membrane stability and neuronal repair, and excessively low cholesterol levels have been linked to unfavorable outcomes in several critical illnesses (28).

Importantly, the CONUT score integrates nutritional, inflammatory, and immune information into a single objective index (15). Therefore, particularly in the acute phase of ischaemic stroke, the CONUT score should not be interpreted solely as a marker of malnutrition, but rather as a composite indicator reflecting nutritional status, systemic inflammation, immune response, and overall disease severity. Critically ill patients with AIS are often exposed to hypercatabolic stress, impaired oral intake, and systemic inflammation simultaneously (29, 30). Under these conditions, poor nutritional status may further aggravate immune dysfunction and organ failure, ultimately leading to increased mortality risk (30). This may partly explain why the prognostic effect of the CONUT score remained significant even after adjustment for disease severity and major clinical confounders.

From a clinical standpoint, our findings indicate that the CONUT score may provide a convenient method for early risk stratification in critically ill patients with AIS. Because the CONUT score is calculated from routinely available laboratory parameters, it can be rapidly obtained during the early stage of ICU admission without additional cost or complex assessment procedures (15). Patients with elevated CONUT scores may benefit from closer monitoring, closer nutritional evaluation, and earlier preventive management for complications. The machine learning model showed stable predictive ability in both internal and external validation cohorts, suggesting its potential as a prognostic tool. However, prospective validation studies are still needed before routine implementation in neurocritical care practice.

This study has several limitations. First, the study used a retrospective observational design, and residual confounding may still exist despite multivariable adjustment. Second, variables with limited missing data were imputed using mean imputation, which may have underestimated data variability and introduced potential bias. Third, the CONUT score was assessed only using baseline data after ICU admission, and longitudinal changes in nutritional status during hospitalization were not analyzed. Fourth, some potentially important variables, such as infarct volume, NIHSS score, and detailed treatment information, were unavailable in the database. Finally, although the model was externally validated using an independent cohort from a tertiary hospital in China, the external cohort was derived from a single center. Differences in clinical practice, ICU organization, nutritional management, and stroke care pathways across healthcare systems may therefore limit the generalizability of our findings. Further prospective multicenter studies are needed to verify its generalizability and potential clinical utility before routine implementation.

## 5 Conclusion

Higher CONUT scores were independently associated with increased short-term and long-term mortality in critically ill patients with AIS. The CONUT score may represent a simple and objective marker for early nutritional assessment and risk stratification in neurocritical care settings. In addition, the CatBoost-based machine learning model demonstrated good predictive performance and external generalizability, suggesting potential value for individualized prognosis evaluation after further prospective validation.

## Data Availability

The raw data supporting the conclusions of this article will be made available by the authors, without undue reservation.
